# Surgical benchmarks, mid-term oncological outcomes, and impact of surgical team composition on simultaneous enbloc robot-assisted radical cystectomy and nephroureterectomy

**DOI:** 10.1186/s12894-021-00839-y

**Published:** 2021-04-28

**Authors:** Stephan Buse, Assen Alexandrov, Elio Mazzone, Alexandre Mottrie, Axel Haferkamp

**Affiliations:** 1 Department of Urology, Alfried Krupp Krankenhaus, Hellweg 100, 45276 Essen, Germany; 2 Department of Urology and Paediatric Urology, University Medical Center, Johannes-Gutenberg-University, Mainz, Germany; 3 Landesklinikum Wiener Neustadt, Vienna, Austria; 4 Division of Oncology/Unit of Urology, URI, IRCCS Ospedale San Raffaele, Milan, Italy; 5 Vita-Salute San Raffaele University, Milan, Italy; 6 Department of Urology, OLV Aalst, Aalst, Belgium; 7 ORSI Academy, Melle, Belgium

**Keywords:** Minimally invasive surgery, Radical cystectomy, Radical nephroureterectomy

## Abstract

**Background:**

Simultaneous urothelial cancer manifestation in the lower and upper urinary tract affects approximately 2% of patients. Data on the surgical benchmarks and mid-term oncological outcomes of enbloc robot-assisted radical cystectomy and nephro-ureterectomy are scarce.

**Methods:**

After written informed consent was obtained, we prospectively enrolled consecutive patients undergoing enbloc radical cystectomy and nephro-ureterectomy with robotic assistance from the DaVinci Si-HD® system in a prospective institutional database and collected surgical benchmarks and oncological outcomes. Furthermore, as one console surgeon conducted all the procedures, whereas the team providing bedside assistance was composed ad hoc, we assessed the impact of this approach on the operative duration.

**Results:**

Nineteen patients (9 women), with a mean age of 73 (SD: 7.5) years, underwent simultaneous enbloc robot-assisted radical cystectomy and nephro-ureterectomy. There were no cases of conversion to open surgery. In the postoperative period, we registered 2 Clavien-Dindo class 2 complications (transfusions) and 1 Clavien-Dindo class 3b complication (port hernia). After a median follow-up of 23 months, there were 3 cases of mortality and 1 case of metachronous urothelial cancer (contralateral kidney).The total operative duration did not decrease with increasing experience (r = 0.174, *p* = 0.534). In contrast, there was a significant, inverse, strong correlation between the console time relative to the total operative duration and the number of conducted procedures after adjusting for the degree of adhesions and the type of urinary diversion(r = -0.593, *p* = 0.02).

**Conclusions:**

These data suggest that en bloc simultaneous robot-assisted radical cystectomy and nephro-ureterectomy can be safely conducted with satisfactory mid-term oncological outcomes. With increasing experience, improved performance was detectable for the console surgeon but not in terms of the total operative duration. Simulation training of all team members for highly complex procedures might be a suitable approach for improving team performance.

*Trial registration*: Not applicable.

**Video Abstract**

**Supplementary Information:**

The online version contains supplementary material available at 10.1186/s12894-021-00839-y.

## Background

The guideline-adherent treatment of bladder and upper urinary tract malignancies implies radical cystectomy and nephroureterectomy [1, 2]. While metachronous urothelial cancer in the lower and upper urinary tract has been reported in a relevant proportion of patients initially diagnosed with urothelial cancer in the upper urinary tract [3] and to a lesser extent in patients initially diagnosed with urothelial cancer in the bladder [4], simultaneous urothelial cancer manifestation in the lower and upper urinary tract affects 2% of patients [5]. Combined lower and upper urinary tract resection is warranted in these patients. While data describing the surgical benchmarks of simultaneous enbloc robot-assisted radical cystectomy and nephro-ureterectomy [6–11] have been reported, information on mid-term oncological outcomes is lacking.

Without the proper training of the whole surgical team (not only console surgeons), the operative duration will unavoidably be prolonged, particularly for complex procedures. A prolonged operative duration has been associated with an increased incidence of postoperative complications in laparoscopic and robotic procedures [12, 13]. In addition, a predictable operative duration is crucial for the efficient scheduling of operating room utilization, as both under- and overscheduling have major economic impacts. Among other factors, the team composition plays a relevant role in the operative duration beyond the learning curve [14–18]. To date, the impact of the surgical team composition on the duration of enbloc robot-assisted radical cystectomy and nephro-ureterectomy, a complex, multistep procedure, has not been explored.

The aims of this manuscript were as follows: (1) to report the surgical benchmarks and postoperative complications; (2) to report mid-term oncological outcomes of simultaneous enbloc robot-assisted radical cystectomy and nephro-ureterectomy at a single centre; and (3) to assess the impact of the team composition (console surgeon vs patient-side surgeon) on the duration of this procedure.

## Methods

### Patient population and study design

After written informed consent was obtained, we enrolled 19 consecutive patients undergoing simultaneous enbloc robot-assisted radical cystectomy and nephro-ureterectomy between 2010 and 2019 at Alfried Krupp Hospital, Essen, Germany, in a prospective institutional database; routine clinical data, including surgical benchmarks, postoperative complications and oncological outcomes of patients on follow-up at our institution, were prospectively entered into the institutional database. This was a retrospective analysis of the data collected in the institutional database. The need for ethics approval for the retrospective analysis of routine clinical data was waived by the local ethics board (Ethik-Kommission, Ärztekammer Nordrhein).

### Surgical team and technique

A single, experienced (over 3000 robot-assisted procedures) console surgeon (SB) performed all procedures. Patient-side surgical assistance was provided by various urology residents based on availability (ad hoc composition). All residents were exposed daily to routine robotic surgery. The surgical nurse team was also assigned ad hoc from a dedicated robotic surgery team of 5 nurses.

We performed all procedures with robotic assistance from the DaVinci Si-HD® system (Intuitive Surgical, Inc., Sunnyvale, US), a four-arm robotic system. We have previously described in detail our technique for simultaneous enbloc robot-assisted radical cystectomy and nephro-ureterectomy [6]. Briefly, with the patient in a 60-degree flank position, we introduced 5 ports for transperitoneal nephro-ureterectomy preparation and prepared the ureter and the renal hilum. Next, the renal vessels were clipped, and the kidney was completely mobilized while maintaining ureter continuity. Cystectomy was then completed with the patient in a supine position using 5 ports, i.e., the system had to be undocked/redocked, and new ports had to be inserted. The patient was positioned in a Trendelenburg position for mobilization and preparation of the seminal vesicles, the base of the prostate and the bladder or the uterus, the ovaries and the bladder. The enbloc specimen (kidney, ureter, bladder, prostate or uterus/ovaries) was removed using a specimen bag through expansion to 6–8 cm of the previous port incision in the lower abdomen in men or transvaginally in women. After reduction of the Trendelenburg position to a tilt of 10 degrees, we performed urinary diversion using an ileal conduit or ureterocutaneostomy (UCST). The video in the online supplements (Additional file 1) shows the surgical technique step by step and provides a video abstract of the results.

UCST was conducted in palliative situations, in patients with severe comorbidities either to limit the operative duration or to avoid bowel resection in patients at high risk for anastomotic complications, or based on patient-specific considerations [6] after a detailed discussion of the benefits and risks with the individual patients prior to surgery.

### Endpoints and data collection

Surgical benchmarks of interest included the total operative duration (incision to skin suture) and console time, blood loss, surgical margins, conversion to open surgery, and postoperative complications ≥ class 2 according to the Clavien-Dindo classification [19]. The endpoints for mid-term outcomes, defined as a follow-up duration >  = 1 year [20], were all-cause death and cancer recurrence.

Preoperatively, we collected data on age, body mass index (BMI), American Society of Anesthesiologists (ASA) classification, and tumour stage. The degree of intrabdominal adhesions was extracted from the surgical reports, where it was recorded as “none”, “minor to moderate” or “extensive” by the surgeon. The institutional convention is to describe the degree of adhesions in the surgical report as “minor to moderate” if adhesiolysis required less than 30 min or as “extensive” if adhesiolysis required more than 30 min.

### Statistical analysis

We described frequency data as counts and percentages and continuous data as the median (interquartile range Q1–Q3) or the mean (standard deviation, SD), as appropriate. Surgical benchmarks, postoperative complications, and mid-term oncological outcomes are reported descriptively.

To assess the impact of the experience of the console surgeon vs that of the patient-side team, we used a partial correlation to quantify the relationship between each console and non-console time by the number of conducted simultaneous enbloc robot-assisted radical cystectomy and nephro-ureterectomy (main independent variable) procedures, controlling by the degree of intraperitoneal adhesions and the type of urinary diversion (UCST vs ileal conduit) as covariables.The console and non-console times are both expressed as percentages of the total operative duration. The limited number of cases prevented the entry of additional covariables, e.g., BMI. SPSS 25 was used for all analyses.

## Results

### Surgical benchmarks and postoperative complications

Nineteen patients (9 women), with a mean age of 73 (SD: 7.5) years and a mean BMI of 27.5 (SD: 5), underwent simultaneous enbloc robot-assisted radical cystectomy and nephroureterectomy. The ASA class was ≥ 3 in 13 patients (68.4%), i.e., indicating a major comorbidity burden. Ten (52.6%) patients had a history of previous abdominal procedures: 5 (26.3%) patients presented minor to moderate intraperitoneal adhesions, and 5 (26.3%) patients presented extensive intraperitoneal adhesions. The bladder tumour stage was pTa/pTis, pT1, and pT2a and prostate pT4a in one patient each, pT3a/b in 6 (31.6%) patients, and pT4a/b in 9 (47.4%) patients. Upper urinary tract tumours consisted of 1 renal oncocytoma and urothelial cancer pTa in 3, pT1 in one, and pT3 in 2 patients. In 12 patients, the indication for the combined procedure was a functionless kidney. Urinary diversion consisted of an ileal conduit in 5 patients and of a UCST in 14 patients in the presence of severe comorbidities, advanced age, or a palliative approach.

Overall, surgery lasted 324 min (SD: 65), with 208 min (SD: 57) of console time. The median estimated blood loss was 220 mL (Q1–Q3: 200–250). There were no cases of conversion to open surgery. The bladder resection margins were positive in 3 patients (1 pT4a and 2 pT4b), and the ureteral resection margins were positive in 1 patient (pT3).

In the postoperative period, 2 patients required transfusion (Clavien-Dindo class 2) and one patient suffered from a port hernia (Clavien-Dindo class 3b).

### Mid-term oncological outcomes

During a median follow-up of 23 months (IQR: 9–42), 1 patient developed metachronous urothelial cancer (contralateral kidney) at 24 months, 1 patient died at 1 month from pulmonary embolism, and 2 patients died from tumour progression, one at 4 and one at 62 months.

At the mid-term, the median estimated creatinine clearance was 44 mL/min (IQR: 36–50), which did not significantly differ from the preoperative clearance (median: 45 mL/min, IQR: 32–62).

### Impact of team composition on operative duration

UCST surgery lasted a mean of 310 min (SD: 68), with a console duration of 204 min (SD: 58). Surgery in patients receiving ileal conduit lasted a mean of 355 min (SD: 69), with a mean console duration of 217 min (SD: 59). The total operative duration did not change with the progression of surgical experience (r = 0.174, *p* = 0.534). On average, the console duration represented 65% (SD:10) of the total operative duration in UCST patients and 60% (SD 10%) in ileal conduit patients. In contrast to the total duration, there was a significant, inverse, strong correlation between the console time relative to the total operative duration and surgical experience (r = − 0.593, *p* = 0.02) after controlling for the degree of adhesions and the type of urinary diversion. Blood loss was not correlated with the progression of surgical experience (Spearman r: − 0.150, *p* = 0.593). The low number of complications prevented any assessment in this regard.

## Discussion

This series of consecutive patients undergoing enbloc robot-assisted radical cystectomy and nephro-ureterectomy suggests that this complex procedure can be conducted with an acceptable operative duration and limited blood loss by an experienced robotic surgeon. Furthermore, the procedure resulted in a limited postoperative complication rate. The mid-term oncological outcomes were in line with the survival estimates for urothelial bladder cancer [21, 22]. As such, these findings support the findings from previous reports [6–11] regarding surgical safety while adding information in terms of mid-term oncological outcomes.

With regard to the team composition, the data suggested a steep improvement in console surgeon performance (expressed as the console duration) with the progression of surgical experience after taking into account the degree of abdominal adhesions. This is in line with previous data reporting that experienced console surgeons have a steep learning curve even for technically demanding procedures, e.g., 20–30 cases for radical cystectomy [23, 24]. As such, these data seem to expand the applicability of a steep learning curve in experienced surgeons to include very rarely performed procedures.

In contrast to the console duration, the total operative duration did not decrease with increasing centre experience with this specific procedure, even after taking into account the degree of adhesions. This fact suggests that while the console surgeon improved his performance, for the patient-side part of the procedure, i.e., positioning, pneumoperitoneum, port insertion, docking and undocking, there was stagnation. We interpret this observation as the result of an ad hoc composition of the surgical team (patient-side surgeon and nurses). Indeed, while the same console surgeon performed all procedures, the patient-side surgeon and surgical nurses were assigned ad hoc from a pool of urology residents and specialized robotic surgical nurses. While exposed daily to routine robot-assisted procedures, such as prostatectomy, the rare confrontation of the team composed ad hoc with this infrequent procedure might have, in our interpretation, prevented increases in the efficiency of the surgical team as a whole and therefore might have negatively affected the total operative duration. Because the total operative duration is a crucial variable for resource utilization and for patient outcomes [12, 13], this issue is of clinical relevance. Simulation training research [25–32] often focuses on the console surgeon, and only recently have virtual simulators integrating the console surgeon and bedside assistants become available. However, considering that a non-negligible part of the operative duration (on average, approximately 1/3 in this cohort) seems to be driven by the patient-side steps of the procedure, training including positioning and port placement/replacement and docking/undocking specific to the procedure [27] might be helpful to improve performance in terms of the total operative duration.

Previous studies have addressed the impact of the team composition regarding the experience of the patient-side assistant [33–35] for different robot-assisted urological procedures. After adjusting for prostate size, the seniority of the bedside assistant did not affect the total operative duration in a retrospective cohort of 106 patients undergoing robot-assisted prostatectomy [33]. We consider that the divergence to our findings is the result of a very different type of procedure: on the one hand, RALP is acommon and routine intervention; on the other hand, en bloc cystectomy and nephro-ureterectomy is a rarely performed, extensive procedure requiring different patient positions, port locations and repeated docking/undocking. Furthermore, the classification of residency by year used in that study might not optimally reflect the actual proficiency level of residents in procedural assistance. In the context of a more complex routine procedure, such as robot-assisted partial nephrectomy, there was a significant association [34] or a strong trend (*p* = 0.051) [35] for an impact of the level of experience of the bedside assistant on the operative duration. Other perioperative benchmarks, e.g., blood loss, warm ischaemia time, and surgical margin status, did not differ [35].

### Limitations

At our centre, we conduct over 400 robot-assisted procedures per year; however, since 2010, a limited number of patients have required simultaneous enbloc robot-assisted radical cystectomy and nephro-ureterectomy. As such, the main limitation of this study is the limited sample size. However, due to the low prevalence of simultaneous urothelial cancer [5], this might reflect the clinical reality even for high-volume centres. The limited sample size prevented adjustment for multiple potentially relevant variables (e.g., T-stage, BMI), and we limited adjustment to the degree of adhesions and the type of urinary diversion. The small sample size also resulted in high variability. We addressed this issue by using relative (percentage of the total operative duration) rather than absolute console and non-console durations. Furthermore, during the study period, the console surgeon had performed a large number of other robot-assisted procedures that may have influenced the reported outcomes on console time improvement; however, this was also the case for the bedside assistants, so this fact is first, reflective of the clinical reality and second, was not differential, i.e., it applied to all members of the surgical team.

## Conclusions

These data suggest that en bloc simultaneous robot-assisted radical cystectomy and nephro-ureterectomy can be safely conducted with satisfactory mid-term oncological outcomes. With increasing experience, improved performance was detectable for the console surgeon but not in terms of the total operative duration. Simulation training of all team members for highly complex procedures might be a suitable approach for improving team performance.

## Data Availability

The datasets generated and/or analysed during the current study are not publicly available because patients were not asked for consent for data sharing. Summary and anonymized data may be available upon justified request.

## Abbreviations

ASA: American society of anesthesiologists
BMI: Body mass index
Q1-Q3: Interquartile range
SD: Standard deviation
UCST: Ureterocutaneostomy
